# The Cerebral Effect of Ammonia in Brain Aging: Blood–Brain Barrier Breakdown, Mitochondrial Dysfunction, and Neuroinflammation

**DOI:** 10.3390/jcm10132773

**Published:** 2021-06-24

**Authors:** Danbi Jo, Byeong C. Kim, Kyung A. Cho, Juhyun Song

**Affiliations:** 1 Department of Anatomy, Chonnam National University Medical School, Hwasun 58128, Jeollanam-do, Korea; 198390@jnu.ac.kr; 2 BioMedical Sciences Graduate Program (BMSGP), Chonnam National University, Hwasun 58128, Jeollanam-do, Korea; kacho@jnu.ac.kr; 3 Department of Neurology, National University Medical School, Gwangju 61469, Korea; byeong.kim7@gmail.com; 4 Department of Biochemistry, Chonnam National University Medical School, Hwasun 58128, Jeollanam-do, Korea

**Keywords:** ammonia, brain aging, blood–brain barrier (BBB) breakdown, neuroinflammation, mitochondrial dysfunction, cognitive decline

## Abstract

Aging occurs along with multiple pathological problems in various organs. The aged brain, especially, shows a reduction in brain mass, neuronal cell death, energy dysregulation, and memory loss. Brain aging is influenced by altered metabolites both in the systemic blood circulation and the central nervous system (CNS). High levels of ammonia, a natural by-product produced in the body, have been reported as contributing to inflammatory responses, energy metabolism, and synaptic function, leading to memory function in CNS. Ammonia levels in the brain also increase as a consequence of the aging process, ultimately leading to neuropathological problems in the CNS. Although many researchers have demonstrated that the level of ammonia in the body alters with age and results in diverse pathological alterations, the definitive relationship between ammonia and the aged brain is not yet clear. Thus, we review the current body of evidence related to the roles of ammonia in the aged brain. On the basis of this, we hypothesize that the modulation of ammonia level in the CNS may be a critical clinical point to attenuate neuropathological alterations associated with aging.

## 1. Introduction

Aging is a substantial global health issue and is markedly increasing in prevalence [1]. People aged over 65 years old are termed “elderly people,” and the aging population is dramatically increasing worldwide because of lower birth rates [2].

Aging changes multiple biochemical and physiological cellular mechanisms, reduces the functions of organs such as the brain [3], and ultimately results in a high risk of neurodegenerative diseases, metabolic disorders, and cancer [4,5,6].

In the central nervous system (CNS), aging causes brain atrophy, poor motor and learning skills, and reduction in attention [7]. As there are many risk factors for the aging process in the CNS, including various metabolites, aged blood vessels, hyperlipidemia, impaired glucose metabolism, excessive reactive oxygen species (ROS) production, and poor energy metabolism [8,9,10], we investigated brain aging from various viewpoints.

Ammonia is a gaseous component generated during metabolism, and high levels of ammonia have been reported to have deleterious effects on cells [11]. During aging, the level of ammonia in both the blood and the CNS is altered, and altered ammonia levels contribute to multiple neuropathological mechanisms, such as cognitive decline [12].

Herein, we review recent evidence on the roles of ammonia in the aged brain, focusing on the breakdown of the blood–brain barrier (BBB), neuroinflammation, and memory function.

## 2. Brain Aging

The surviving global human population is aging rapidly and is increasingly made up of people aged over 65 years old [2]. Aging is a biochemical and physiological process directly related to life span, the risk of cancer, and neurodegenerative diseases [4,5,6]. Aging leads to the reduction in the functional capacity of diverse organs including the brain [3]. In particular, aging is considered the main cause of cognitive decline and decreasing attention [7]. The aged brain has a reduced volume of brain tissue [13,14] and shrinkage of gray matter that is considered indicative of neuronal cell death [15].

One study demonstrated that age accelerates memory loss through synaptic dysfunction [16]. Another study showed that aging results in a decrease in learning ability, reduction in motor coordination, and reduced sensitivity to sensory perception [17]. Moreover, impaired stress response speed, hearing loss, and a decrease in word retrieval ability are also associated with the aged brain [18].

The gray and white matter in the brain shrink with aging, while brain cerebral ventricles expand, ultimately leading to cognitive dysfunction [19,20,21]. A previous study showed that the structure of the brain changes with age, and the volume of brain areas, such as the frontal cortex, are also reduced with age, thus causing a reduction in cognitive function [22].

Mechanically, brain aging is associated with neuronal mitochondrial dysfunction and, subsequently, DNA damage and impaired energy homeostasis, such as abnormal ATP consumption, compared with normal brain cells [9,23,24]. An increase in mitochondrial membrane permeability and mitochondrial fragmentation in the aged brain leads to neuronal cell death and an increased risk for neurodegenerative diseases [25,26,27].

During aging, neurons are exposed to conditions of oxidative stress, leading to the production of reactive oxygen species (ROS) and nitric oxide (NO), resulting in elevated intracellular Ca^2+^ levels [28]. One study reported that the aging brain cortex is exposed to excessive NO-induced oxidative stress [10] and showed moderate levels of accumulation of the lipid peroxidation product 4-hydroxynonenal (HNE) [8]. Another study reported that the aged brain attenuates the activity of lysosomes and proteasomes and shows impaired autophagy [29]. Additionally, the aged brain shows an abnormal secretion of neurotransmitters including glutamate and serotonin, impaired activity of neurotransmitter receptors such as AMPA and NMDA receptors, and impaired Ca^2+^ influx into CNS cells [30]. In addition, brain aging causes aberrant neural connectivity through impaired GABAergic neuronal signaling, glutamatergic neuronal signaling, dopaminergic neuronal signaling, cholinergic neuronal signaling, and serotonergic neuronal signaling, as well as excitatory imbalances [31,32,33]. Several studies have also reported brain aging as a chronic inflammatory disease and have associated it with increased levels of pro-inflammatory cytokines such as tumor necrosis factor-α (TNF-α), interleukin-6 (IL-6), and IL-1β [34,35].

Numerous studies have shown that the BBB breaks down in the aged brain, leading to neurodegenerative diseases such as Alzheimer’s disease (AD) [36]. BBB leakage leads to an increase in neuronal cell death, excessive iron accumulation, and imbalances in nutrient support to CNS cells [37]. Recently, some studies have shown that metabolic morbidities, including obesity, dyslipidemia, insulin resistance, and dysregulated glucose metabolism, are critical issues affecting the aged brain, suggesting that metabolic imbalances lead to severe cognitive decline [38,39,40] and result in the onset of dementia [41].

Brain aging is associated with a diverse range of diseases including metabolic syndromes, such as obesity and diabetes, and neurodegenerative diseases such as dementia [42,43,44]. Epidemiological studies have reported that elderly people show lower cognitive performance accompanied by various metabolic problems such as hypertension and dyslipidemia [45,46].

As mentioned earlier, aging alters the structure and function of the brain through an increase in neuroinflammation, breakdown of the BBB, mitochondrial dysfunction, acceleration of neuronal cell death, and severe memory loss. To identify appropriate prevention and treatment methods for brain aging, further studies and multilateral clinical approaches are necessary for the next generation.

## 3. Roles of Ammonia in the Aged Brain

Ammonia exists in two forms (ammonia gas [NH_3_] and ion [NH_4_^+^]) and is a crucial gaseous element in organic metabolism; however, excessive levels lead to cellular toxicity [11,47,48]. During normal organic metabolism, approximately 17 g of ammonia is produced in the human adult body daily [49].

Excessive ammonia levels in the brain can be due to impaired glucose metabolism resulting from liver failure [50]. Additionally, another source of excessive ammonia in the brain is adenosine-3-monophosphate (AMP) deaminase that can convert to ammonia [51]. During aging, AMP deaminase is decreased; in addition, elevated ammonia levels are found in the aged AD brain [51]. Moreover, elevated ammonia levels are involved in the reduction of glutamine synthesis [52].

Increases in ammonia levels in the brain lead to memory loss through synaptic dysfunction and imbalance of neurotransmitters, contributing to the onset of hepatic encephalopathy [53]. In the setting of liver failure, ammonia rapidly accumulates in the brain, compared with that during normal conditions [54], ultimately contributing to impaired glucose metabolism, poor synaptic transmission, and lack of glutamate secretion [55,56,57]. One study reported that high levels of ammonia are found in the aged neurodegenerative brain, such as the AD brain [58]. Overall, alterations in ammonia levels in the aged brain are very important indicators of neuropathological changes. However, identification of the detailed mechanisms of ammonia in the aged brain requires further study.

### 3.1. BBB Breakdown and Ammonia in Brain Aging

The BBB, as a selective semipermeable borderline that prevents imprudently solute’s passing in the circulating blood into the brain, is composed of endothelial cells of the capillary wall, astrocytes end feet, and pericytes stuck in the capillary basement membrane [59].

BBB breakdown is a critical feature in brain aging and changes the cerebral microvascular environment in the brain, resulting in cognitive decline [60,61]. Some researchers have reported that high levels of ammonia in the brain change the microvascular structure and damage the BBB structure while simultaneously altering the structure of astrocytes and neurons [62]. Recent studies have demonstrated that hyperammonemia causes cerebral edema and BBB breakdown [62,63,64].

Pericyte, as a component of BBB, could control biochemical functions of BBB by regulating the formation of tight junction proteins and controlling vesicle trafficking in endothelial cells [65]. Additionally, one study using the pericyte-deficient mouse model demonstrated that pericyte could help microcirculation by suppressing brain capillary perfusion and maintain BBB structure against brain damage [66].

**Figure 1:**
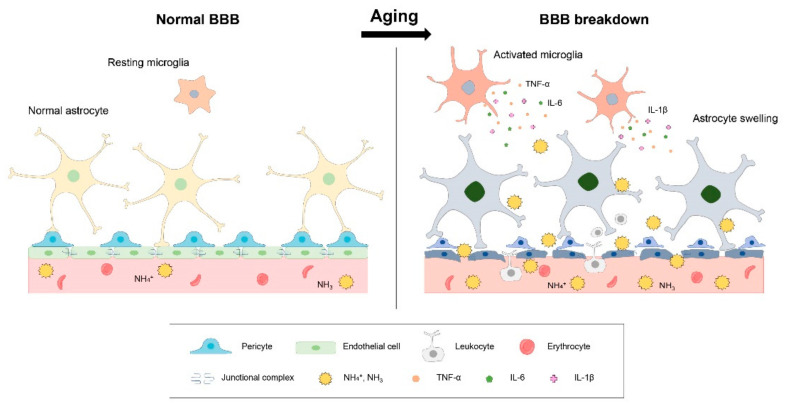
BBB breakdown and neuroinflammation in aged brain.

Aquaporins (AQPs) are water transport proteins. They are also linked to the transport of ammonia across cell membranes and are associated with BBB permeability [67,68]. In particular, AQP3, 4, 7, 8, and 9 are membrane proteins related to ammonia permeability [69,70,71,72,73]. Furthermore, the ammonia NH_3_ permeability pathway associated with BBB breakdown is related to the H+-coupled NH_3_ cotransporter (SLC4A11) [74], SLC12A2 [75], and an increase in p21 expression [76]. Several studies have shown that hyperammonemia aggravates BBB leakage by degrading the tight-junction proteins mediated by activation of matrix metalloproteinases (MMP)3 and MMP9 [77,78] (Figure 1).

Although the breakdown of the BBB is a feature of the aged brain, elevated ammonia levels aggravate brain aging by accelerating BBB disruption. Hence, inhibiting the breakdown of the BBB by modulating ammonia levels may be a good clinical approach for alleviating neuropathologies in elderly people.

In the aged brain, blood–brain barrier (BBB) is gradually collapsed by the degradation of the tight-junction proteins and the damage of brain endothelial cells and astrocytes. Ammonia accelerates severe BBB disruption in the aged brain through astrocyte swelling and the boosting of inflammatory responses in the aged brain.

### 3.2. Neuroinflammation and Ammonia in Brain Aging

In CNS, microglia and astrocytes are cells that regulate inflammatory responses and maintain BBB homeostasis and the brain’s immune system [79,80,81]. During aging, chronic neuroinflammation and immune system impairments occur, causing cognitive dysfunction and increasing the risk of dementia [82,83]. Studies have demonstrated that high levels of ammonia accelerate the excessive production of NO and ROS, as well as the expression of pro-inflammatory cytokines in the cerebral cortex, cerebellum, and striatum [84,85,86,87]. In addition, elevated levels of ammonia decrease the activity of antioxidant enzymes, ultimately leading to an increase in cell death [88,89].

One study suggested that high levels of ammonia reduce phagocytosis in glia and induce apoptosis through nuclear factor-kappa B (NF-κB) signaling [90]. Furthermore, high levels of ammonia result in excessive accumulation of glutamine in astrocytes, triggering astrocyte swelling and leading to apoptosis [91].

Astrocyte swelling contributes to brain edema and intracranial pressure increase as well as cell death [57,92,93,94]; it involves several inflammatory signaling molecules such as NF-κB [95] (p. 53), [96] (p. 38), mitogen-activated protein kinase (p38 MAPK), nuclear factor erythroid-derived 2-like 2 (Nrf2), and heme oxygenase-1 (HO-1) [97,98]. Under conditions of increased ammonia levels, microglia and astrocytes are highly activated and produce pro-inflammatory cytokines [99,100] including TNF-α, IL-1β, and IL-6 [101,102], leading to severe inflammation (Figure 1).

Despite the progression of neuroinflammation in the aged brain, the increased ammonia concentration in the brain enhances brain aging and induces a variety of neuropathological problems. The regulation of ammonia levels in the aged brain may be beneficial for attenuating neuroinflammatory responses, which might be helpful for maintaining cognitive function in elderly people.

### 3.3. Mitochondria Dysfunction and Ammonia in Brain Aging

**Figure 2:**
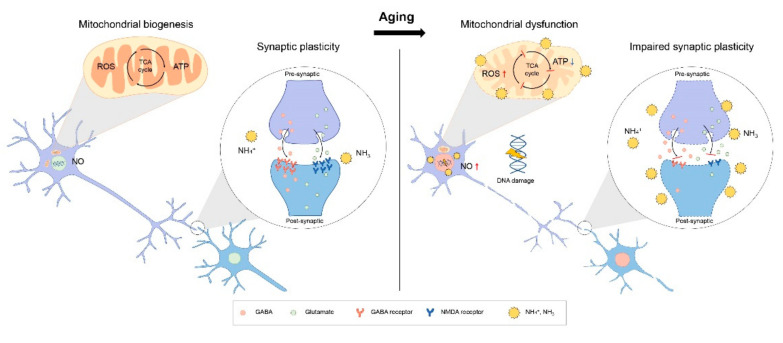
Mitochondria dysfunction and impaired synaptic plasticity in aged brain.

Mitochondria are centers for the production of chemical energy in the form of ATP in CNS cells [103]. With aging, CNS cells show mitochondrial dysfunction, such as impaired mitochondrial biogenesis, reduced mitochondria membrane potential, and decreased mitochondrial density [103]. Some studies have reported a reduction in mitochondrial enzymatic activity in the aged brain, compared with the normal brain [104,105] (Figure 2).

In the aged brain, mitochondrial function in CNS cells is not normal, and subsequently, mitochondrial dysfunction leads to poor energy metabolism in CNS cells. Neurons are damaged with age, and this damage in neurons is boosted by ammonia toxicity. In the aged brain, neuron produces more amount of ROS and NO, and neuron showed DNA fragment by high ammonia level. Synaptic plasticity is influenced by neurotransmitter secretion and the expression of neurotransmitter receptors in neurons. Ammonia encourages impaired synaptic function in the aged brain, leading to memory loss.

High levels of ammonium contribute to energy metabolism by inhibiting the tricarboxylic acid (TCA) cycle in neurons and glia [106], leading to a decrease in ATP production in mitochondria [107,108]. Previous studies have demonstrated that excessive ammonia levels lead to impaired mitochondrial membrane potential [109,110] and loss of ATP in cultured astrocytes [111]. Recent studies have implicated that high concentration-ammonia-induced toxicity leads to impaired mitochondrial function [112] and also impairs the activity of key enzymes in the mitochondria, leading to abnormal energy metabolism in the brain [113] (Figure 2).

Although the mitochondrial function is influenced by brain aging, high concentrations of ammonia in the brain accelerate brain aging through the deterioration of mitochondrial dysfunction. To maintain mitochondrial function and energy metabolism in the brain, ammonia levels in the aged brain need to be regulated.

### 3.4. Cognitive Decline and Ammonia in Brain Aging

Almost all elderly people complain of memory loss and pathological problems in language ability [114,115]. Older people suffer from reductions in semantic memory [116], procedural memory [117], episodic memory [118], and working memory [119]. Some studies have demonstrated that hyperammonemia leads to atrophy of the brain cortex and demyelination, leading to cognitive decline and cerebral palsy [120,121]. Under hyperammonemic conditions, NMDA receptors are reduced in the brain [122]. In addition, another study reported that overdose administration of ammonium chloride attenuates the expression of two NMDA receptor subunits in the hippocampus and is associated with cognitive function [123]. One study showed that elevated ammonia levels aggravate energy metabolism and neurite outgrowth, leading to memory dysfunction [124]. Administration of excessive ammonium chloride leads to the abnormal secretion and uptake of neurotransmitters such as dopamine and GABA [125,126]. High levels of ammonia are known to be associated with the critical causes of neuropsychiatric problems [127].

Several studies have reported that ammonia-induced inflammatory responses lead to memory loss [128,129] and motor dysfunction [130]. Furthermore, one study demonstrated that excessive ammonia inhibits the induction of long-term potentiation (LTP), which is considered a cognitive function mediated by the GABA receptor [131] (Figure 2). Most elderly people across the world complain of memory loss; thus, the regulation of high ammonia levels is needed to slow memory deterioration in these people.

## 4. Discussion

Herein, we reviewed the roles and mechanical functions of ammonia in the aged brain from diverse perspectives. Ammonia levels in the brain increase with age and are involved in alterations in synaptic function, neuroinflammation, and memory function. High levels of ammonia trigger rapid and severe BBB breakdown, neuroinflammation, mitochondrial dysfunction, and cognitive decline in the aged brain. Thus, adjusting the ammonia levels in the brain may be a therapeutic solution to inhibit neuropathological symptoms.

Clinical trials for high ammonia toxicity-induced neuropathological problems include the use of NMDA receptor antagonists, NO inhibitors, and acetyl-l-carnitine [120,132,133]. However, there is currently a lack of understanding regarding the mechanisms of action of ammonia in the aged brain. To treat the neurological problems caused by ammonia, further study with respect to ammonia’s effects in the aged brain is required. Thus, we suggest that the modulation of ammonia levels in the aged brain may be key to preventing and treating various neurological pathologies.

## Data Availability

Not applicable.
